# The Inclusion of Rights of People with Disabilities and Women and Girls in Water, Sanitation, and Hygiene Policy Documents and Programs of Bangladesh and Cambodia: Content Analysis Using EquiFrame

**DOI:** 10.3390/ijerph18105087

**Published:** 2021-05-11

**Authors:** Nathaniel Scherer, Islay Mactaggart, Chelsea Huggett, Pharozin Pheng, Mahfuj-ur Rahman, Adam Biran, Jane Wilbur

**Affiliations:** 1 International Centre for Evidence in Disability, London School of Hygiene & Tropical Medicine, London WC1E 7HT, UK; islay.mactaggart@lshtm.ac.uk (I.M.); jane.wilbur@lshtm.ac.uk (J.W.); 2 WaterAid Australia, Melbourne, VIC 3002, Australia; chelsea.huggett@wateraid.org.au; 3 WaterAid Cambodia, Phnom Penh 12207, Cambodia; pharozin.pheng@wateraid.org.au; 4 WaterAid Bangladesh, Dhaka 1213, Bangladesh; mahfujurrahman@wateraid.org; 5 Environmental Health Group, London School of Hygiene & Tropical Medicine, London WC1E 7HT, UK; adam.biran@lshtm.ac.uk

**Keywords:** people with disabilities, women and girls, water, sanitation and hygiene, WASH, policy, rights, Bangladesh, Cambodia, low- and middle-income countries

## Abstract

People with disabilities and as women and girls face barriers to accessing water, sanitation, and hygiene (WASH) services and facilities that fully meet their needs, especially in low- and middle-income countries. Women and girls with disabilities experience double discrimination. WASH policies should support and uphold the concepts of disability and gender inclusion, and they should also act as a guide to inform WASH programs and service delivery. Using a modified version of the EquiFrame content analysis tool, this study investigated the inclusion of 21 core concepts of human rights of people with disabilities and women and girls in 16 WASH policy documents and seven end-line program reports from Bangladesh and Cambodia. Included documents typically focused on issues of accessibility and neglected wider issues, including empowerment and support for caregivers. The rights of children and women with disabilities were scarcely focused on specifically, despite their individual needs, and there was a disconnect in the translation of certain rights from policy to practice. Qualitative research is needed with stakeholders in Bangladesh and Cambodia to investigate the inclusion and omission of core rights of people with disabilities, and women and girls, as well as the factors contributing to the translation of rights from policy to practice.

## 1. Introduction

### 1.1. Inequitable WASH

Access to water, sanitation, and hygiene (WASH) is a fundamental human right, as recognized in the 2010 United Nations General Assembly Resolution 64/292 and the Sustainable Development Goals (SDGs) [1,2]. Despite global progress over the last 30 years, estimates indicate that 2.1 billion people do not have access to safe drinking water, and 4.5 billion lack safe sanitation services [3,4]. As with all SDGs, Goal 6: Clean water and sanitation, follows the guiding principle of “leave no one behind” [5]. However, barriers to accessing WASH services disproportionately affect certain groups, especially in low- and middle-income countries (LMICs), including people with disabilities, and women and girls [4,6]. This inequitable access to WASH impacts their health, livelihood, and education opportunities [4].

Recent multi-country analyses reported that the majority of people with disabilities could access the same WASH facilities as other household members, but they frequently required assistance to do so and often faced difficulties [7]. One survey of 20,000 households in Bangladesh found that 47% of people with disabilities found it difficult to access sanitation facilities without coming into contact with feces, whilst 79% were unable to collect water. Furthermore, people with disabilities experience stigma and discrimination when accessing WASH [8]. In Uganda, people with disabilities were not allowed to use communal water points as they were seen to be dirty, and their impairment was thought to be contagious [9]. Such stigma and discrimination can result in people with disabilities being excluded from participating in WASH decision processes as well as the planning, development, and implementation of services and programs [10]. People with disabilities also report instances of physical, verbal, and sexual abuse when accessing public WASH facilities [11].

Inequitable WASH is a gendered issue, and women and girls are disproportionally affected by lack of access due to biological and cultural issues [4]. Limited access to WASH resources and facilities leads to poorer physical and psychological health outcomes in women and children, including infection and disease (such as soil-transmitted helminth infections and schistosomiasis), which are associated with maternal and newborn mortality [12]. Moreover, women and girls bear the brunt of unpaid WASH responsibilities in households and communities. In a systematic review of 59 studies from 30 countries, estimates of women as primary carriers of water ranged from 61% to 79%, and it is a role associated with poorer health, pain, and musculoskeletal disorders [13]. In a systematic review of 76 studies, women and girls in LMICs also reported restrictions on menstrual hygiene management, resulting from inadequate infrastructure and the economic environment, as well as cultural expectations and stigma [14]. As a result, women and girls reported feelings of shame and distress when menstruating. Women and girls are also at risk of abuse and violence when using WASH facilities and services [15]. Evidence from Kenya found that women and girls were at risk of physical and sexual violence when fetching water or when using WASH facilities, especially at night [16].

These barriers and challenges to disability and gender inclusive WASH intersect, and women and girls with disabilities experience double discrimination, placing them at higher risk of violence, exclusion, and exploitation. For instance, in Cambodia, women with disabilities have reported exclusion from community meetings, making it difficult for them to learn about WASH and health management [17]. In a systematic review of 22 studies from 14 countries, women with disabilities and caregivers reported challenges and difficulties in menstrual hygiene management, including limited training and information, and a lack of appropriate menstrual hygiene materials for individuals with a physical impairment [18]. Through qualitative research in Malawi, menstruation was found to be a source of shame, discomfort, and worry for women with disabilities, and adolescents with disabilities were reported to drop out of school when beginning menstruation [19]. In Nepal, information on menstrual hygiene management was often withheld from women with intellectual disabilities because of perceived limitations in understanding [20]. As a result, some showed their menstrual blood and hygiene products to others and were abused for doing so, whilst others became frightened and withdrawn during menstruation.

### 1.2. Disability Inclusive Development

A definition of ‘inclusive WASH’ does not exist in peer-reviewed literature, although a number of non-governmental organizations (NGOs) have formulated their own. An inclusive approach addresses barriers to accessing and using WASH services and facilities. Key pillars of inclusive WASH include participation of users in decision-making and access for all.

International frameworks and collaborations exist to support investment in disability and gender-inclusive WASH projects, programs and policies, including a directive from the United Nations Children’s Fund (UNICEF) for investment in accessible and inclusive WASH, which focused on the SDGs principle of “leave no one behind” [21]. Furthermore, the United Nations Convention on the Rights of Persons with Disabilities (UNCRPD) provides evidence and guidance for new policy frameworks that aim to promote disability-inclusive societies [22].

Whilst the Overseas Development Institute (ODI) has reported global progress in tackling discriminatory legal and policy frameworks, the implementation of disability-inclusive programs has been slow in LMICs, especially with regard to women and girls with disabilities, suggesting a disconnect between policy and practice [23]. Reasons reported for this include: a lack of coordination to enable cross-sectoral programing and accountability; limited trained service providers; and few evaluations to provide evidence of what works.

In 2015, the Rural Water Supply Network recognized that “water user committee members, government health educators, and village heads need simple and clear guidance or manuals on how to promote inclusive WASH” [24]. Responding to this need, we aim to develop evidence-based guidance for governments in LMICs on implementing disability-inclusive WASH at scale. This guidance will provide information on including disability within WASH policy and recommendations on translating policy commitments into practice. With inequalities in WASH disproportionately affecting women and girls, and women and girls with disabilities, the guidance will offer direction on support for this population.

To formulate this guidance, we are conducting research in Bangladesh and Cambodia, evaluating contributing factors to implementing inclusive WASH in LMICs. As a first step in guidance development, we have examined the inclusion of people with disabilities, and women and girls, in WASH policy documents and programs in Bangladesh and Cambodia. Bangladesh and Cambodia were chosen as research sites because of government commitment to the rights of people with disabilities in WASH service provision. Assessments in these countries have highlighted positive steps taken by each government toward a disability-inclusive approach in WASH, but reported gaps remain [25,26]. For example, in Cambodia, the Disability Action Council developed the ‘National Disability Strategic Plan 2014–2018’, after ratifying the UNCRPD. Although this has helped improve the policy framework on the rights of people with disabilities and provides guidance on accessible public health infrastructure, it does not make mention of inclusive WASH specifically. It has been some time since each country review, and this study provides further opportunity to assess advances in disability-inclusive development and inclusive WASH. Both countries operate a decentralized system, in which the national government is responsible for policy formation and local government implementation.

### 1.3. Aims and Objectives

This study aimed to examine the extent to which WASH policy documentation in Bangladesh and Cambodia include information on the rights of people with disabilities, and women and girls, and the degree to which policy commitments have been translated into program implementation.

The objectives of the study were as follows: (i) identify the information on the rights of people with disabilities, and women and girls, included in WASH policy documents of Bangladesh and Cambodia; and (ii) understand the extent to which implemented WASH programs integrate these rights into practice.

## 2. Materials and Methods

We conducted a content analysis of WASH policy documents and program reports from Bangladesh and Cambodia.

### 2.1. Selection of Documents

#### 2.1.1. Policy Documents

Policy documents were identified through an online literature search and through interaction with key actors involved in influencing national WASH policy and practice in Bangladesh, Cambodia, and Australia. We also reviewed research articles in which the WASH policies of each country were analyzed, in search of additional policy documents, for example, ‘An analysis of Water Policies and Strategies of Bangladesh in the Context of Climate Change’, from Hadi in 2019 [27]. WASH documents were included if they were specific to WASH or its component parts (water, sanitation, and hygiene) and developed by the governments of Bangladesh or Cambodia, for national use. Eligible document types included policies, action plans, strategic objectives, and national guidelines, which were either strategic or operational in nature. Legislative documentation was not included nor were documents provided by regional or local authorities.

#### 2.1.2. Program Reports

Information on implemented WASH programs was sought through an online review and search of national and international NGOs and other entities working on WASH in each country, as well as searches by teams in Bangladesh and Cambodia. In order to apply an assessment of realized WASH implementation and outcomes, rather than those intended, we included only end-line program reports produced by the implementing organization or an external party. Protocols, inception reports, or mid-line reviews were not included. To be eligible, end-line reports must have included information on the program components and realized outputs. The program could have focused on implementing services or facilities for any aspect of WASH, for any population, at either a national, regional, or local level.

All policy and program documents were required in English. Where documents were not available in English, local partners translated documents from the local language. There was no date restriction applied, although policies needed to be the most current version in use by the national government.

### 2.2. Content Analysis

We adapted and used the EquiFrame content analysis tool to evaluate the rights of people with disabilities, and women and girls, included in the WASH policy documents and programs of Bangladesh and Cambodia. EquiFrame is a policy analysis framework that has been designed to assess a policy against its inclusion of 21 core concepts of human rights deemed essential for universal, equitable, and accessible health services [28,29]. This tool is not typically used to examine program reports, but we have applied the same logic. In addition to evaluating the inclusion of 21 core concepts of human rights, the tool is designed to assess the inclusion of 12 vulnerable groups, including people with disabilities, female-headed households, and mothers of young children (<5). With our focus being on the rights of people with disabilities, and women and girls in WASH, we adapted the tool to evaluate inclusion of the 21 core concepts for these two target groups only (people with disabilities, and women and girls) each potentially vulnerable to exclusion from WASH.

EquiFrame provides key language and key questions for each of the 21 core concepts. We refined these to reflect the rights of people with disabilities, and women and girls, in the context of WASH (Table 1), by mapping each concept against the ‘human right to water and sanitation’, which was adopted by the United Nations General Assembly in 2010 [1]. During adaptation, we sought advice and feedback from the EquiFrame development team [29].

### 2.3. Equiframe Scoring

Reference to a core concept, in relation to the rights of people with disabilities, or women and girls, received a score of 1 to 4:

1. The concept was mentioned;
2. The concept was mentioned and explained;
3. Specific policy actions were identified to address the concept;
4. Intention to monitor the concept was expressed.

In end-line program reports, the scoring of 1 and 2 remained the same, with criteria altered slightly for scores of 3 and 4:

3. Specific actions were taken in the program to address the concept;
4. Steps to monitor the concept were taken in the program.

Scores of 3 and 4 indicate a reference to the core concept is operational and actionable, and the reference is deemed ‘high quality’.

For each document, two independent reviewers (NS and JW) coded each reference to a core concept against the scoring criteria. References, in this context, typically refer to a single sentence or short paragraph. For example, the following sentence taken from the Cambodian policy document ‘National Guidelines on WASH for Persons with Disabilities and Older People’ would be included as a single reference and scored 3 under the concept of *Participation*: “Programs should include persons with disabilities and older people in the planning, implementation, and evaluation of activities where possible, including the participation of DPO [Disabled People’s Organization] and OPA [Older People’s Association] representatives, and representatives from NGOs that already have expertise in working with these groups”.

Scores were applied separately to references of rights of people with disabilities, and women and girls. Overlapping references (i.e., with regard to women and girls with disabilities) were scored twice, under each group. Any discrepancy in score was discussed by the reviewers until a consensus was reached.

Summary statistics were generated for each document individually and then combined across both countries under policy and program documentation. Statistics relevant to the rights of people with disabilities, and women and girls, are presented separately. Data were aggregated across the two countries to help us learn lessons on commonly endorsed and neglected concepts, to take forward into guideline development, rather than focus on the strengths and limitations of each individual country approach. To derive these data, the following statistics were calculated.

#### 2.3.1. Scoring across All Documents

1. **Core concept reference:** the proportion (%) of all references to a core concept across policy and program documents, split by disability and gender. This was calculated for Bangladesh and Cambodia individually, and aggregated together;
2. **Average score:** the average score across all references to a concept, providing insight into the commitment of included information.

#### 2.3.2. Scoring per Document

1. **Core concept coverage:** Each document was examined with respect to the proportion of the 21 core concepts referenced at least once;
2. **Core concept quality:** The proportion of references to all 21 core concepts scored 3 or 4 (i.e., stating a specific action or an intention to monitor that action).

#### 2.3.3. Document Excerpts

Excerpts from included documents were extracted and are presented as an illustrative example of a single reference, in the context of total scoring. The concept scored is presented with the excerpt.

#### 2.3.4. Ethical Approval

The study was approved by the Ethics Committee of the London School of Hygiene & Tropical Medicine (17679), the National Ethics Committee for Health Research in Cambodia (042), and the Bangladesh Medical Research Council (BMRC/NREC/2019–2022/608).

## 3. Results

For clarity and ease of interpretation, we have presented the results and summary statistics under two thematic areas: disability and gender inclusion.

### 3.1. Policy Documents

A total of 16 WASH policy documents, 10 from Bangladesh and six from Cambodia, were included in the analysis. Table 2 presents the proportion of references (across all policy documents) and average score attributed to each of the 21 core concepts. Table S1 details the core concept coverage (i.e., the proportion of core concepts referenced at least once) and the proportion of references that are scored 3 or 4 for each included policy document.

#### 3.1.1. Core Concept Reference and Coverage: Disability Inclusion

Information related to disability inclusion was most commonly provided with regard to the rights of *Access* (21% of all references) and *Individualized services* (17%).

*“Include sanitation businesses in disability awareness raising and encourage universal accessible design principles—emphasizing the benefits and usability of the whole community throughout a persons’ life cycle” (National Guidelines on WASH for Persons with Disabilities and Older People: Cambodia; Scored Access)*

The concepts of *Contribution* and *Accountability* were not referenced in any policy documentation from either country. The concepts of *Liberty*, *Autonomy*, *Privacy*, *Family resource*, *Family support*, *Prevention,* and *Efficiency* were referenced relatively infrequently (≤1%) compared with other concepts. Most (62%) of the references were scored 3 or 4 (‘high quality’).

Of the 333 core concept references, 90% regarded people with disabilities as a broad group, with 4% made in relation to adults with disabilities only, 4% to children with disabilities, and 2% to the needs of adults and children with disabilities as individual groups. Only 7% of references referred to women with disabilities.

Across both countries, WASH policy documents referenced 21% of core concepts, on average, in relation to the rights of people with disabilities (Table S1). Five policies (three in Bangladesh, two in Cambodia) did not reference a single core concept in relation to disability inclusion, and the overarching national WASH policy of neither country included reference to any core concept.

#### 3.1.2. Core Concept Reference and Coverage: Gender Inclusion

Under gender inclusion, *Individualized services* (23%) was the most commonly referenced concept. The concepts of *Liberty*, *Family resource,* and *Family support* were not included in any policy document, whilst *Autonomy*, *Privacy,* and *Accountability* were referenced relatively infrequently (≤1%). Half (50%) of the concept references were scored 3 or 4 (‘high quality’).

*“Ensure hand pumps and water containers are women- and girl-friendly, and are designed in ways that minimize the time spent collecting water” (Operational Guidelines for WASH in Emergencies: Bangladesh; scored Individualized services).*

More than one-third (37%) of the 346 references provided information relevant to females broadly, with no mention of age group, whilst 35% focused on women only and 10% on girls. Meanwhile, 18% of references referred to women and girls as individual groups.

In contrast to disability inclusion, all policy documents referenced at least one core concept in regard to gender inclusion. On average, each document referenced 34% of core concepts.

### 3.2. Program Documents

Seven end-line program reports were included, of which five were implemented in Bangladesh.

#### 3.2.1. Core Concept Reference and Coverage: Disability Inclusion

Capacity building (27%) was the most frequently referenced concept in relation to disability inclusion (Table 3).

*“The disability-friendly latrines installed by the project were used as a demonstration to the local government and service providers, such as the municipal and sub-district governments, Department of Public Health and Engineering, and other NGOs.” (ADD International—Improved Sanitation for Women and Children with Disabilities living in extreme poverty in Bangladesh; scored Capacity building)*

The concepts of *Protection from harm*, *Liberty*, *Autonomy*, *Privacy*, *Contribution*, *Family support*, *Cultural responsiveness*, *Accountability,* and *Prevention* were not mentioned in any program report. Most (90%) of references to the core concepts were scored at 3 or 4 (‘high quality’).

Of the 91 references, 66% were made in relation to people with disabilities broadly, 4% were focused on adults with disabilities, 12% were focused on children with disabilities only, and 18% were focused on both adults and children with disabilities as individual groups. Meanwhile, 19% of references were made in relation to women with disabilities.

Program reports from Bangladesh and Cambodia referenced 14% of core concepts, on average, in relation to disability inclusion (Table S2). Of the seven reports included, three did not reference a single concept in relation to disability inclusion.

#### 3.2.2. Core Concept Reference and Coverage: Gender Inclusion

Under the theme of gender inclusion, *Capacity building* (18%) was again most commonly referenced, with *Non-discrimination* (14%), *Individualized services* (13%), and *Participation* (11%) referenced relatively frequently (≥10%), compared with other concepts. *Coordination of services*, *Liberty*, *Privacy*, *Family support,* and *Accountability* were not referenced in any program document, whilst *Autonomy*, *Contribution*, *Family resource,* and *Prevention* were referenced relatively infrequently (≤1%). Most (85%) references scored 3 or 4 (‘high quality’).

Of 190 references, 26% were made with respect to females broadly, while 38% were made in relation to women only, and 24% were made with respect to girls. 12% recognized women and girls as individual groups.

*“Include a detailed gender baseline in relation to WASH at communities and schools for being able to identify gender related needs, actions and indicators” (Child Rights Foundation: Improving Hygiene and Sanitation of Cambodia Rural Schools and Communities; scored Non-discrimination and Quality)*

With so few references in the program reports of Cambodia, the summary indices shown in Table 3 are predominantly driven by the findings from Bangladesh.

All core concepts were referenced at least once, in relation to gender inclusion. On average, program reports referenced 33% of concepts, with regard to the rights of women and girls.

### 3.3. Mapping Policy to Program

In Cambodia, there was disparity in the inclusion of rights of people with disabilities between policy and program documents, with program reports referencing just two concepts. The program reports of Bangladesh demonstrated commitment to a greater breadth of rights of people with disabilities, although these did not always correspond to policy documents. For example, *Capacity building* (28%), the most commonly referenced concept in program documentation, is referenced infrequently (2%) in policy.

This trend is seen again under concepts related to gender inclusion. In Cambodia, the program documents reference few of the 21 core concepts and do not once reference *Individualized services*, which is the most commonly referenced concept (20%) in the country’s WASH policy documents. Programs in Bangladesh demonstrated a greater breadth of core concepts with regard to the rights of women and girls, with a number receiving similar proportional representation in policy and program documents—*Participation* (11%), for example. As with the theme of disability inclusion, *Capacity building* (19%) was more commonly included in programs, compared to policy documents (2%).

## 4. Discussion

As a first step in developing guidance on inclusive WASH policy and practice in LMICs, this study examined the inclusion of 21 core concepts of human rights of people with disabilities, and women and girls, in WASH policy documents and programs from Bangladesh and Cambodia.

When analyzed using EquiFrame, the UNCRPD included 95% of core concepts, highlighting the suitability of the tool and the core concepts to an assessment of disability inclusion [30]. In relation to the rights of people with disabilities, the WASH policy documents examined included only 21% of EquiFrame’s 21 core concepts, on average. Both Bangladesh and Cambodia have committed to the SDGs, and the guiding principle of “leave no one behind”, and WASH policy documents should be including information relevant to a greater breadth of rights of people with disabilities, in order to support disability-inclusive development. The proportion of concepts included in relation to the rights of women and girls was higher: 34% on average. This trend is also seen in the program reports: 14% (disability inclusion) vs. 33% (gender inclusion). This may reflect a better understanding of WASH for women and girls or a greater commitment of support to this group, given their larger demographic representation and contributions to WASH at the household level. It may also reflect the longer history of gender inclusion in policy, compared to disability; the UNCRPD was adopted in 2006, whereas the Convention on the Elimination of All Forms of Discrimination against Women (CEDAW) was adopted in 1979 [22,31]. Two-thirds of the core concepts were still typically not included in the policy documents with regard to gender inclusion, which is an omission that should be addressed.

### 4.1. Accessible and Appropriate WASH Services and Facilities

In realizing disability-inclusive WASH, barriers to accessing services and facilities must be alleviated, whether they be physical, attitudinal, or institutional [10]. Reflecting this, *Access* is the most commonly referenced concept in the UNCRPD, and this is observed in the WASH policy documents of Bangladesh and Cambodia (21%) reviewed in this study [29]. However, *Access* is not frequently referenced in program reports (5%), despite its stated value in policy documents and the UNCRPD. The reasons for this are unclear and will be important to explore in future qualitative research. As well as *Access*, information on *Individualized services* is frequently included in policy and program documentation, in recognition of the specific WASH needs of people with disabilities (17% and 18%, respectively), and women and girls (23% and 13%, respectively). Although relatively few references are made to women with disabilities overall, when done so, *Individualized services* is recognized most frequently (18% in policies and 35% in programs).

However, there are a number of factors that contribute to accessibility and individualized services, which have been neglected. For example, *Entitlement* is rarely included in either policy (5%) or program (2%) documentation, despite financial burden and poverty being major barriers to WASH access for people with disabilities in LMICs [32]. EquiFrame’s core concepts do not exist in isolation of one another, and this highlights the need to recognize the relationships between each and provide guidance that incorporates all.

### 4.2. Shifting Unequal Power Dynamics

The concept and right of *Participation* is included relatively frequently in WASH policy documentation with regard to disability and gender inclusion (11% each), reflecting the drive for greater empowerment of women and girls and inclusion of people with disabilities in WASH (and other) systems. In the disability sector, this includes the movement of “Nothing About Us, Without Us”, which advocates the principle that people with disabilities know what is best for them and their community and must be valued as contributors to the policies, programs, and services that affect their lives [33,34].

That being said, the majority of references across policy documents and programs are focused on WASH infrastructure (i.e., concepts of *Access* and *Individualized services*) and other than *Participation*, very few references relate to rights and concepts of empowerment of people with disabilities, and women and girls, such as *Autonomy and Contribution*. Empowerment and participation reduce stigma and discrimination, which can, in turn, promote further empowerment and participation, helping to break the cycle of exclusion [35,36,37].

Empowerment must also reflect the capacity of people with disabilities, and women and girls, in the implementation and management of WASH services and facilities. Women and people with disabilities are often excluded from positions of leadership in WASH, whether that be in policy development, WASH management committees, or program implementation teams. Supporting these groups to exercise their capability and capacity is needed to achieve a systems change toward inclusive WASH [38]. *Capability-based services* is included in policy documentation for both groups (6% disability inclusion and 8% gender inclusion), to some extent, and program reports indicate a greater degree of support in practice for people with disabilities (16%). To further promote successful and sustainable implementation of inclusive WASH will require training, capacity building, and support for management committees, government leadership, service providers, and civil society organizations [10].

### 4.3. WASH and Health

WASH is an important determinant of individual health and protection from disease, a notable example being its importance in interrupting the transmission of the coronavirus disease 2019 (COVID-19) [39]. Women and girls may also face additional health concerns due to WASH burdens and duties, such as carrying water. However, *Prevention* of health conditions associated with WASH is a right rarely included in policy or program documentation (≤2%). As demonstrated in a 2019 systematic review, there is a dearth of evidence on WASH interventions and their effect on infections in health care settings in LMICs, and this lack of research may contribute to a limited focus and understanding of *Prevention* [40]. Further research on WASH interventions, with specific focus on the needs of people with disabilities, and women and girls, would support evidence-based approaches in policy and practice.

### 4.4. Reducing the Risk of Violence Associated with WASH

Despite being vulnerable to abuse when using WASH facilities, information on *Protection from harm* is rarely included in relation to people with disabilities, in either policy documents (3%) or programming (0%). However, it is referenced more frequently in policy documentation, in relation to women and girls (12%), which is a positive finding, although few specific details are given on the protection of women with disabilities. Safety from abuse is of the utmost importance in WASH provision. The Sanitation and Hygiene Applied Research for Equity (SHARE) consortium has developed a practitioner’s toolkit on ensuring safe and accessible WASH services, which may aid governments and service providers in providing safe WASH services for people with disabilities, and women and girls [41].

### 4.5. Recognizing the Role of Caregivers

It is important to note few references toward *Family resource* and *Family support* in either policy or program documents, which is not reflective of the key role that family members and caregivers provide in supporting people with disabilities in WASH practice. Many family members take on informal caregiving duties for people with disabilities, and this responsibility is often placed on women in particular. Family members and caregivers without support may be unaware of effective hygienic WASH practices on topics such as menstrual health and hygiene, limiting their ability to best support their family member manage their menstruation hygienically and comfortably [42]. Moreover, results from Nepal and Malawi document that additional WASH-based tasks placed upon caregivers of people with disabilities result in psychological distress and feelings of isolation [19,20]. Support, training, and guidance for families and caregivers is needed to ensure inclusive WASH provision.

### 4.6. Target Groups

As with the broader population, WASH is an important public health concern for child health, impacting survival and development. Conditions such as diarrhea, worms, and dehydration (all linked to poor WASH) are associated with long-term negative outcomes in mortality, physical growth, and cognitive, motor and language functioning [12,43]. Evidence from a number of countries, including Bangladesh, demonstrates the role of WASH provision and intervention in promoting survival, growth, and child development [44,45,46]. Further evidence estimates that improvements in sanitation practices account for just under 10% of the decline in child mortality, between 1990 and 2015 [47].

Despite the importance of WASH for children, just 4% of references in included policy documents target children with disabilities. As well as the impact on survival and development, inadequate and inaccessible WASH facilities contribute to children with disabilities not attending school, and they can increase a child’s vulnerability to abuse [10]. Children with disabilities are a marginalized and vulnerable group, facing a number of challenges in school and community life, and the very little reference of this group in policy contributes to their continued exclusion [48]. There is an argument that children with disabilities are included in references to people with disabilities more broadly (90%), and although correct, there is a clear need for specific policy direction for this group, given the number of important, individualized considerations for them in WASH provision. The proportion of references targeting children with disabilities is considerably higher in program documentation (26%). One contributing factor to this higher proportion may be the analysis of the ADD International-run program, ‘Improved Sanitation for Women and Children with Disabilities living in extreme poverty in Bangladesh’, in which children with disabilities are a targeted beneficiary and referenced frequently.

In contrast, girls are well represented across both policy documents (28%) and programs (36%). WASH is a vital consideration for girls, impacting educational outcomes and menstrual health and hygiene as well as survival and development [49]. Strong proportional representation of girls in policy and practice is needed to advance gender inclusion, and the results of this study indicate that girls are represented in Bangladesh and Cambodia to a similar extent as adult women, which is an encouraging finding.

Relatively few references are made to the rights of women with disabilities. This is higher in programs (19%) than in policy (7%), but as with children with disabilities, this is driven by the assessment of the ADD International program, in which WASH support for women with disabilities is a core focus. Women with disabilities are disproportionately affected by inequalities in WASH, and for the policies of Bangladesh and Cambodia to omit specific considerations for this group is a major oversight. It is important that this group not be forgotten in WASH service provision.

### 4.7. Policy to Practice

The observed disparities in relative inclusion of rights of people with disabilities, and women and girls, between policy documents and program reports may suggest a disconnect between policy development and program implementation. In Cambodia, only two policy documents were available prior to the first program report published in 2015, of which one contained no information on disability-inclusive rights, and the other was focused on rural populations. This may account for the limited reference to the rights of people with disabilities in the program reports of Cambodia.

Across programs, *Capacity Building* is highly valued, with regard to disability (27%), and gender inclusion (18%), but this is less the case in policy documents (9% and 3%, respectively). Limited knowledge and skills in appropriate practices are often a barrier to disability-inclusive WASH, and capacity building is a core need across the sector for WASH leadership and service implementers, as well as communities and households [10,50]. Greater inclusion of this concept in programming may reflect the limited personnel capable of implementing disability and gender-inclusive WASH, and thus the need to incorporate capacity strengthening schemes into program delivery, to ensure successful implementation. This supports findings from ODI, who reported limited capacity as a primary reason for the slow implementation of disability-inclusive programs [23]. The policy documents of Bangladesh and Cambodia, and future guidance, may demand greater reference to *Capacity building*, as the situation amongst implementing organizations appears to indicate a need.

Further, *Coordination of services*, *Efficiency*, and *Accountability* are rarely referenced in the policy and program documentation, despite their importance to sustained disability and gender-inclusive WASH provision. Again, these areas are reported by ODI to be factors toward the slow implementation of disability-inclusive programming in LMICs, and they appear to be a needed focus in guidance on disability-inclusive WASH, if countries are to implement inclusive WASH at scale [23].

The findings of this study will inform qualitative research in both countries, in which we can explore and identify the factors that facilitate and disrupt the implementation of disability-inclusive WASH policies and programs in more detail. We will be interviewing policy-makers, service providers, women and men with disabilities, and caregivers. Disparities in policy and program (for example, with regard to the inclusion of information relevant to *Access* and *Capacity Building*) will be discussed. Lessons learned from this content analysis and the qualitative research will help inform our future guidance development. This inclusive WASH guidance will complement the new operational guide on leaving no one behind, developed by the United Nations Sustainable Development Group to support Member States in reaching the furthest behind first, across all rights enshrined in the SDGs [51].

### 4.8. Limitations

Our inclusion criteria for program reports was broad, to help capture as many program reports as possible. Those published by the same implementation team or organization may be subject to information bias, depending on the specific purpose and intended audience of the end-line report. Our original aim was to include both planning and end-line reports, but these documents were rarely publicly available. In only including publicly available end-line reports, we have missed the opportunity to assess the inclusion of rights in programs that did not produce a final evaluation or have these accessible online. We tried to mitigate this by contacting program implementers directly and by working with teams in Bangladesh and Cambodia to source documents not readily available through online searches. Despite our broad inclusion criteria, just two program reports were collected from Cambodia, limiting the interpretation of findings from this country. We conducted the search for program reports with the help of in-country teams, but with our focus typically on larger NGOs and organizations known to be operating on WASH projects, reports may have been missed from grassroots organizations. A more systematic search strategy of bibliographic databases would have been preferred, but with very few program reports indexed on these platforms, we believed our strategy of searching organizations directly offered the most appropriate methodology. However, there is the risk of selection bias, as described. With each country operating a decentralized system, it would also have been beneficial to analyze local or regional WASH policies, to expand our analysis, although these should always be guided by the national policies included in this review.

In interpretation of the data, it is important to acknowledge that many of the concepts may have been referenced in the documents, without a specific focus on people with disabilities, or women and girls. Concepts may have been included more broadly, under a general, all-encompassing language that applies to the entire population. Although information pertinent to these concepts should be covered for people with disabilities, and women and girls, specifically, there may be relevant information for these populations that is not captured in this study.

## 5. Conclusions

Our findings have highlighted the inclusion and neglect of core concepts of human rights of people with disabilities, and women and girls, in WASH policy documents and programs of Bangladesh and Cambodia using EquiFrame. Information relevant to accessibility was commonly included, but greater emphasis is needed toward concepts of empowerment, family support, and sustainable service provision. Specific guidance on the rights of children and women with disabilities is limited and needed in WASH policy and programming. Programs did not reflect the same rights as endorsed in policy documents, and the reasons for this should be explored further in qualitative research. Other countries would benefit from conducting similar analysis to identify gaps in their WASH policies, of which to address.

## Figures and Tables

**Table 1:** The 21 core concepts of EquiFrame, adapted to the rights of people with disabilities, and women and girls, in the context of WASH.

No.	Core Concept	Key Question	Key Language
1.	Non-discrimination	Does the policy support the rights of people with disabilities and women/girls with equal opportunity in receiving WASH services?	People with disabilities and women/girls are not directly or indirectly discriminated against within the WASH system
2.	Individualized services	Does the policy support the rights of people with disabilities and women/girls with individually tailored WASH services to meet their needs, choices, and impairments?	People with disabilities and women/girls receive specific, appropriate, and effective WASH services. For people with disabilities, this includes reasonable adjustments made/supported, when necessary. For women/girls, this may include services specific to menstrual health and hygiene
3.	Entitlement	Does the policy indicate entitlements for people with disabilities and women/girls (e.g., respite grant or reduced user fee), and how they may qualify for specific benefits relevant to them?	People with disabilities and women/girls who have limited resources are entitled to some services free of charge or at a sliding scale tariff, especially if in unpaid work
4.	Capability-based services	Does the policy recognize the capabilities of people with disabilities and women/girls?	For instance, programs including peer support, mentoring, and group advocacy. People with disabilities and women/girls are meaningfully represented in WASH committees. For people with disabilities, programs may be implemented by Organizations of Persons with Disabilities (OPDs)
5.	Participation	Does the policy support the right of people with disabilities and women/girls to participate in the decisions that affect their lives and enhance their empowerment?	People with disabilities and women/girls can exercise choices and influence decisions affecting their life. Consultation may include planning, development, implementation, and evaluation
6.	Coordination of services	Does the policy support assistance of people with disabilities and women/girls in accessing services from within a single provider system (inter-agency) or more than one provider system (intra-agency) or more than one sector (inter-sectoral)?	People with disabilities and women/girls know how services should interact where inter-agency, intra-agency, and inter-sectoral collaboration is required. This includes coordination between health services, schools, households, and public places, with regard to WASH. Additional coordination opportunities include the WASH sector with the private sector, civil society, and rights groups
7.	Protection from harm	Does the policy outline that people with disabilities and women/girls are to be protected from harm during their interaction with WASH and related services?	People with disabilities and women/girls are protected from harm during their interaction with WASH services and health-related systems, as well as from families and the community who may have negative attitudes about WASH for people with disabilities and women/girls (e.g., topics such as menstrual hygiene)
8.	Liberty	Does the policy support the right of people with disabilities and women/girls to be free from unwarranted physical or other confinement?	People with disabilities and women/girls are protected from unwarranted physical or other confinement while in the custody of the service system/provider. This includes at home and a healthcare service
9.	Autonomy	Does the policy support the right of people with disabilities and women/girls to consent, refuse to consent, withdraw consent, or otherwise control or exercise choice or control over what happens to them?	People with disabilities and women/girls can express “independence” or “self-determination”. For instance, a person with an intellectual disability will have recourse to an independent third party regarding issues of consent and choice. Or, as another example, a husband is not to make decisions for his wife
10.	Privacy	Does the policy address the need for information regarding people with disabilities and women/girls to be kept private and confidential?	Information regarding people with disabilities and women/girls need not be shared among others
11.	Integration	Does the policy promote the use of mainstream services by people with disabilities and women/girls?	People with disabilities and women/girls are supported to use the WASH services that are provided for the general population
12.	Contribution	Does the policy recognize that people with disabilities and women/girls can be productive contributors to society?	People with disabilities and women/girls make a meaningful contribution to society and the WASH sector
13.	Family resource	Does the policy recognize the value of the family members of people with disabilities and women/girls in addressing WASH needs?	The document recognizes the value of family members of people with disabilities and women/girls as a resource for addressing WASH needs
14.	Family support	Does the policy recognize individual members of people with disabilities and women/girls may have an impact on the family members requiring additional support from WASH services?	Caring for persons with disabilities and women/girls may impact other family members (e.g., mental health), such that these family members themselves require support
15.	Cultural responsiveness	Does the policy ensure that services respond to the beliefs, values, gender, interpersonal styles, attitudes, cultural, ethnic or linguistic, aspects of the person, as well as personal safety and dignity?	(i) People with disabilities and women/girls are consulted on the acceptability of the service provided; (ii) Hygiene facilities, goods, and services are respectful of ethical principles and culturally appropriate, i.e., respectful of the culture of people with disabilities
16.	Accountability	Does the policy specify to whom, and for what, services providers are accountable?	People with disabilities and women/girls have access to internal and independent professional evaluation or procedural safeguard. Law/regulations provide mechanisms that ensure complaints are effectively heard and there are clear systems for people to lodge these complaints. Judicial bodies are available to resolve conflicts, for both public and private institutions
17.	Prevention	Does the policy support people with disabilities and women/girls in seeking primary, secondary, and tertiary prevention of health conditions associated with WASH?	Includes WASH-related illnesses and details on how people with disabilities and women/girls can seek primary, secondary, and tertiary prevention of health conditions. For example, Trachoma, Soil-Transmitted Helminths—intestinal worms, Lymphatic Flariasis, Leprosy, urinary tract infections
18.	Capacity building	Does the policy support the capacity building of health workers and of the system that they work in addressing WASH needs of people with disabilities and women/girls?	Includes awareness raising among communities and families on disability and on the specific issues/barriers facing people with disabilities and women/girls
19.	Access	Does the policy support people with disabilities and/or women/girls—physical, economic, and information access to WASH services?	People with disabilities and women/girls have accessible and safe WASH services within, or in the immediate vicinity, of household, health, and educational institution, public institutions, and workplace. All information must be understandable and in an appropriate format
20.	Quality	Does the policy support quality services to people with disabilities and women/girls through evidence-based and professionally skilled practice? Does the policy promote innovation in WASH services for people with disabilities and women/girls (e.g., technology)?	People with disabilities and women/girls are assured that services are based on best practice/evidence and support innovative strategies/technology
21.	Efficiency	Does the policy support efficiency by providing a structured way of matching WASH system resources with service demands in addressing WASH needs of people with disabilities and women/girls?	WASH services are sustainable for people with disabilities and women/girls. Services will be available at times of financial crisis and will ensure appropriate technology choices. Contracts with providers take into account operation and maintenance, and funds from donors are sustainable

**Table 2:** Proportion of total references and average score across concept, in the policy documents of Bangladesh and Cambodia (“-“ denotes no reference to a core concept).

		Bangladesh				Cambodia				Total			
		Disability (*n* = 122)		Gender (*n* = 247)		Disability (*n* = 211)		Gender (*n* = 99)		Disability (*n* = 333)		Gender (*n* = 346)	
		% Refs.	Av. Score	% Refs.	Av. Score	% Refs.	Av. Score	% Refs.	Av. Score	% Refs.	Av. Score	% Refs.	Av. Score
1.	Non-discrimination	7%	1.6	6%	1.6	8%	2.9	8%	2.0	8%	2.4	7%	1.2
2.	Individualized services	31%	2.1	23%	2.3	10%	2.6	20%	3.1	17%	1.9	23%	2.2
3.	Entitlement	7%	2.3	2%	2.3	4%	2.6	4%	2.3	5%	2.4	3%	2.3
4.	Capability-based services	2%	3.0	7%	2.6	9%	2.7	7%	2.6	6%	2.8	8%	2.6
5.	Participation	6%	2.4	11%	2.6	13%	2.8	9%	3.0	11%	2.7	11%	2.7
6.	Coordination of services	2%	2.7	4%	2.5	6%	2.7	-	-	5%	2.7	3%	2.5
7.	Protection from harm	7%	2.6	12%	2.7	1%	3.0	10%	2.9	3%	2.6	12%	2.0
8.	Liberty	1%	2.0	-	-	-	-	-	-	<1%	2.0	0%	0.0
9.	Autonomy	1%	1.0	1%	1.7	-	-	-	-	<1%	1.0	1%	1.3
10.	Privacy	1%	3.0	1%	3.0	-	-	-	-	<1%	3.0	1%	3.0
11.	Integration	4%	1.6	3%	1.6	4%	2.5	10%	2.4	4%	2.2	5%	1.9
12.	Contribution	-	-	3%	1.9	-	-	3%	2.0	-	-	3%	1.9
13.	Family resource	-	-	-	-	1%	2.5	-	-	1%	2.5	0%	0.0
14.	Family support	-	-	-	-	1%	2.0	-	-	1%	2.0	0%	0.0
15.	Cultural responsiveness	5%	2.3	4%	2.6	1%	2.0	-	-	3%	2.2	3%	2.4
16.	Accountability	-	-	1%	1.5	-	-	2%	2.0	-	-	1%	1.3
17.	Prevention	1%	1.0	3%	1.8	-	-	-	-	<1%	1.0	2%	0.4
18.	Capacity building	2%	1.7	2%	1.8	12%	2.8	5%	3.0	9%	2.7	3%	2.4
19.	Access	15%	2.7	6%	2.8	24%	3.0	10%	3.1	21%	2.7	8%	1.9
20.	Quality	7%	2.5	6%	2.5	6%	3.0	10%	3.1	6%	2.7	7%	2.1
21.	Efficiency	1%	1.0	2%	1.8	1%	3.5	1%	4.0	1%	2.7	2%	1.9
	Total	100%		100%		100%		100%		100%		100%	

**Table 3:** Proportion of total references and average score across concept, in the program documents of Bangladesh and Cambodia; ^(^“-“ denotes no reference to the core concept).

		Bangladesh				Cambodia				Total			
		Disability (*n* = 89)		Gender (*n* = 176)		Disability (*n* = 2)		Gender (*n* = 14)		Disability (*n* = 91)		Gender (*n* = 190)	
		% Refs.	Av. Score	% Refs.	Av. Score	% Refs.	Av. Score	% Refs.	Av. Score	% Refs.	Av. Score	% Refs.	Av. Score
1.	Non-discrimination	1%	4.0	13%	3.3	-	-	36%	3.0	1%	4.0	14%	3.2
2.	Individualized services	18%	3.7	14%	3.6	-	-	-	-	18%	3.7	13%	3.6
3.	Entitlement	2%	3.0	3%	3.4	-	-	-	-	2%	3.0	3%	3.4
4.	Capability-based services	17%	3.9	10%	3.8	-	-	-	-	16%	3.9	9%	3.8
5.	Participation	4%	3.5	11%	3.7	-	-	7%	4.0	4%	3.5	11%	3.7
6.	Coordination of services	7%	3.7	-	-	-	-	-	-	7%	3.7	-	-
7.	Protection from harm	-	-	3%	3.8	-	-	-	-	-	-	3%	-
8.	Liberty	-	-	-	-	-	-	-	-	-	-	-	-
9.	Autonomy	-	-	1%	3.0	-	-	-	-	-	-	1%	3.0
10.	Privacy	-	-	-	-	-	-	-	-	-	-	-	-
11.	Integration	2%	4.0	5%	2.6	50	4.0	14%	3.5	3%	4.0	6%	2.7
12.	Contribution	-	-	1%	3.0	-	-	-	-	-	-	1%	3.0
13.	Family resource	2%	2.5	1%	2.0	-	-	-	-	2%	2.5	1%	2.0
14.	Family support	-	-	-	-	-	-	-	-	-	-	-	-
15.	Cultural responsiveness	-	-	3%	3.6	-	-	-	-	-	-	3%	3.6
16.	Accountability	-	-	-	-	-	-	-	-	-	-	-	-
17.	Prevention	-	-	1%	4.0	-	-	-	-	-	-	1%	4.0
18.	Capacity building	28%	3.6	19%	3.8	-	-	-	-	27%	3.6	18%	3.8
19.	Access	6%	3.2	10%	3.5	-	-	7%	3.0	5%	3.2	9%	3.4
20.	Quality	8%	4.0	6%	3.6	50%	3.0	36%	3.0	9%	3.9	8%	3.4
21.	Efficiency	4%	3.3	2%	4.0	-	-	-	-	4%	3.3	2%	4.0
	Total	100%		100%		100%		100%		100%		100%	

## Data Availability

Further information on the data presented in this study is available on request from the corresponding author.

## Supplementary Materials

The following are available online at https://www.mdpi.com/article/10.3390/ijerph18105087/s1, Table S1: Core concept coverage across the policy documents of Bangladesh and Cambodia; Table S2: Core concept coverage across the program reports of Bangladesh and Cambodia.
