# Supplementary material for: The Inclusion of Rights of People with Disabilities and Women and Girls in Water, Sanitation, and Hygiene Policy Documents and Programs of Bangladesh and Cambodia: Content Analysis Using EquiFrame
Source: Int J Environ Res Public Health. 2021 May 11;18(10):5087. doi: 10.3390/ijerph18105087 (PMC8151976; doi:10.3390/ijerph18105087)
Supplement: Supplementary file 1 [file ijerph-18-05087-s001.zip › ijerph-1185989-SI.pdf]

Table S1. Core concept coverage across the policy documents of Bangladesh and Cambodia

Policy	Publication year	Disability inclusion		Gender inclusion	
		Core concept coverage (%)	Core concept high quality (%)	Core concept coverage (%)	Core concept high quality (%)
**Bangladesh**					
National Policy for Safe Water Supply & Sanitation	1998	-	-	24%	83%
National Water Policy	1999	-	-	29%	0%
National Standards of Water, Sanitation and Hygiene for Schools in Bangladesh	2010	10%	75%	24%	83%
National Strategy for Water and Sanitation Hard to Reach Areas	2011	-	-	5%	0%
Sector Development Plan	2011	48%	12%	57%	9%
National Hygiene Promotion Strategy for Water Supply and Sanitation Sector	2012	10%	25%	57%	12%
National Strategy for WASH and Sanitation	2014	43%	36%	43%	32%
Operational Guidelines for WASH in Emergencies	2017	52%	69%	71%	80%
National Strategy for WASH in Healthcare Facilities 2019-2023	2019	19%	75%	48%	30%
Pro Poor Strategy for WASH in Bangladesh	2020	19%	17%	24%	50%
		**25%**	**44%**	**48%**	**47%**
**Cambodia**					
National Policy on Water Supply and Sanitation	2003	-	-	10%	20%
National Strategy for Rural Water Supply, Sanitation and Hygiene	2011	38%	50%	57%	38%
Minimum Requirement Guidelines on WASH in Schools (WinS)	2016	10%	100%	14%	86%
National Guidelines on Wash for Persons with Disabilities and Older People	2017	67%	78%	38%	83%
National Guidelines for Water, Sanitation and Hygiene in Healthcare Facilities	2018	19%	79%	14%	100%
National Action Plan Rural Water Supply, Sanitation and Hygiene 2019-2023	2019	-	-	33%	93%
		**22%**	**77%**	**28%**	**70%**

Table S2. Core concept coverage across the programme reports of Bangladesh and Cambodia

Policy	Publication year	Disability inclusion		Gender inclusion	
		Core concept coverage (%)	Core concept high quality (%)	Core concept coverage (%)	Core concept high quality (%)
**Bangladesh**					
BRAC: Water, Sanitation and Hygiene: Nine years of scale and innovation in Bangladesh	2015	-	-	71%	96%
WASHplus: Assessing Water, Sanitation, and Hygiene (WASH) in Southwestern Bangladesh	2016	10%	100%	10%	100%
Department of Public Health Engineering: Bangladesh Rural Water Supply and Sanitation Project	2018	-	-	38%	100%
Bangladesh Red Crescent Society: CDI2 WASH Program - Bangladesh	2018	33%	90%	38%	100%
ADD International: Improved Sanitation for Women and Children with Disabilities living in extreme poverty in Bangladesh	2018	48%	91%	43%	88%
		**18%**	**94%**	**40%**	**97%**
**Cambodia**					
Child Rights Foundation: Improving Hygiene and Sanitation of Cambodia Rural Schools and Communities	2015	-	-	10%	100%
Plan International: A Retrospective Review of Phase 1 of CRSHIP	2017	10%	100%	24%	100%
		**5%**	**100%**	**17%**	**100%**
